# Association of Early Tolvaptan Treatment and Length of In Hospital Stay in Elderly Patients with Acute Decompensated Heart Failure

**DOI:** 10.31662/jmaj.2024-0050

**Published:** 2024-09-20

**Authors:** Sho Suzuki, Kazuhiro Kimura, Nozomu Yoda, Aya Fuchida, Yusuke Kanzaki, Takuya Maruyama, Naoto Hashizume, Ayako Kozuka, Hirohiko Motoki, Kumiko Yahikozawa, Koichiro Kuwahara

**Affiliations:** 1 Department of Cardiovascular Medicine, Shinshu University School of Medicine, Matsumoto, Japan; 2 Department of Cardiovascular Medicine, Minaminagano Medical Center, Shinonoi General Hospital, Nagano, Japan

**Keywords:** Tolvaptan, Heart failure, Hospital stay, Elderly

## Abstract

**Introduction:**

Long hospital stay is associated with high costs and poor quality of life in elderly patients with heart failure (HF). This study aimed to investigate the association of early administration of tolvaptan with length of hospital stay among elderly patients with HF.

**Methods:**

The cohort included elderly patients (age ≥ 75 years) admitted to Shinonoi General Hospital between July 2016 and December 2018 with a primary diagnosis of acute decompensated HF treated with tolvaptan. Patients who died during hospitalization, patients who had acute coronary syndrome, patients who required treatment in the intensive care unit, and patients who had already taken tolvaptan before admission were excluded. Patients were divided into two groups according to the median duration of admission to tolvaptan administration: those who received tolvaptan within 1 day (24 h) after admission (early treatment group) and those for whom tolvaptan was prescribed after 1 day (24 h) or more from hospitalization (add-on group). We compared the length of hospital stay between the two groups and investigated the relationship between early tolvaptan administration and length of hospital stay.

**Results:**

Of 110 enrolled patients (median age 85 years), 56 (51%) received tolvaptan within 1 day (24 h) after admission. The median length of hospital stay was 22 [14-35] days. The length of hospital stay was significantly shorter in the early treatment group (16 [11-22] days vs. 30 [21-46] days, p < 0.001). On multivariable regression analysis, early tolvaptan was associated with shorter hospital stay after adjusting for age, sex, serum creatinine, B-type natriuretic peptide, continuous dobutamine, and whether they live alone (partial regression coefficient −16.213, p < 0.001). Linear regression analysis showed a positive relationship between time of tolvaptan administration and length of hospital stay (R^2^ = 0.564, p < 0.001).

**Conclusions:**

Early tolvaptan administration was associated with reduced length of hospital stay in elderly HF.

## Introduction

Improvements in cardiovascular survival rates and progressive aging of the population have led to an increase in elderly patients with heart failure (HF) ^(1), (2), (3)^. It can be expected that the number of elderly patients with HF will continue to increase in Japan. Due to the high cost of inpatient treatment for HF, the condition represents a major burden on the public health system and, consequently, has a considerable economic impact. Furthermore, extended hospital stays could reduce the activity of daily living (ADL) and quality of life (QOL) of elderly patients with HF. Considering these facts, shortening the length of hospital stay for elderly patients with HF could benefit patients and reduce the economic impacts of the condition.

Tolvaptan, a vasopressin receptor 2 antagonist ^(4)^, is used to treat volume overload in patients with HF when an adequate response is not achieved with other diuretics ^(5)^. Since loop diuretics have several negative impacts such as decreasing glomerular filtration rate ^(6)^ and stimulation of neurohumoral indicators ^(7)^, tolvaptan is sometimes used soon after their hospitalization in acute HF ^(8)^. However, still in many cases, additional administration of tolvaptan is considered after loop diuretics resistance is identified, often several days after admission, and this could lead to the requirement for longer inpatient treatment. Several studies have demonstrated the beneficial effects of early administration of tolvaptan in patients with decompensated HF ^(9), (10), (11)^. A recent study reported that early administration of tolvaptan (within 4 days of admission) significantly shortened the length of hospital stay compared with delayed administration of tolvaptan (over 5 days after admission) ^(12)^. These studies indicate the possibility that the timing of tolvaptan administration after admission should be earlier than is currently considered. In addition, the effect of tolvaptan against elderly patients is not well established. Against this background, this study aimed to identify the association between early tolvaptan treatment and length of hospital stay in elderly patients with acute decompensated HF in a retrospective cohort study.

## Materials and Methods

### Study design

**Figure 1.**
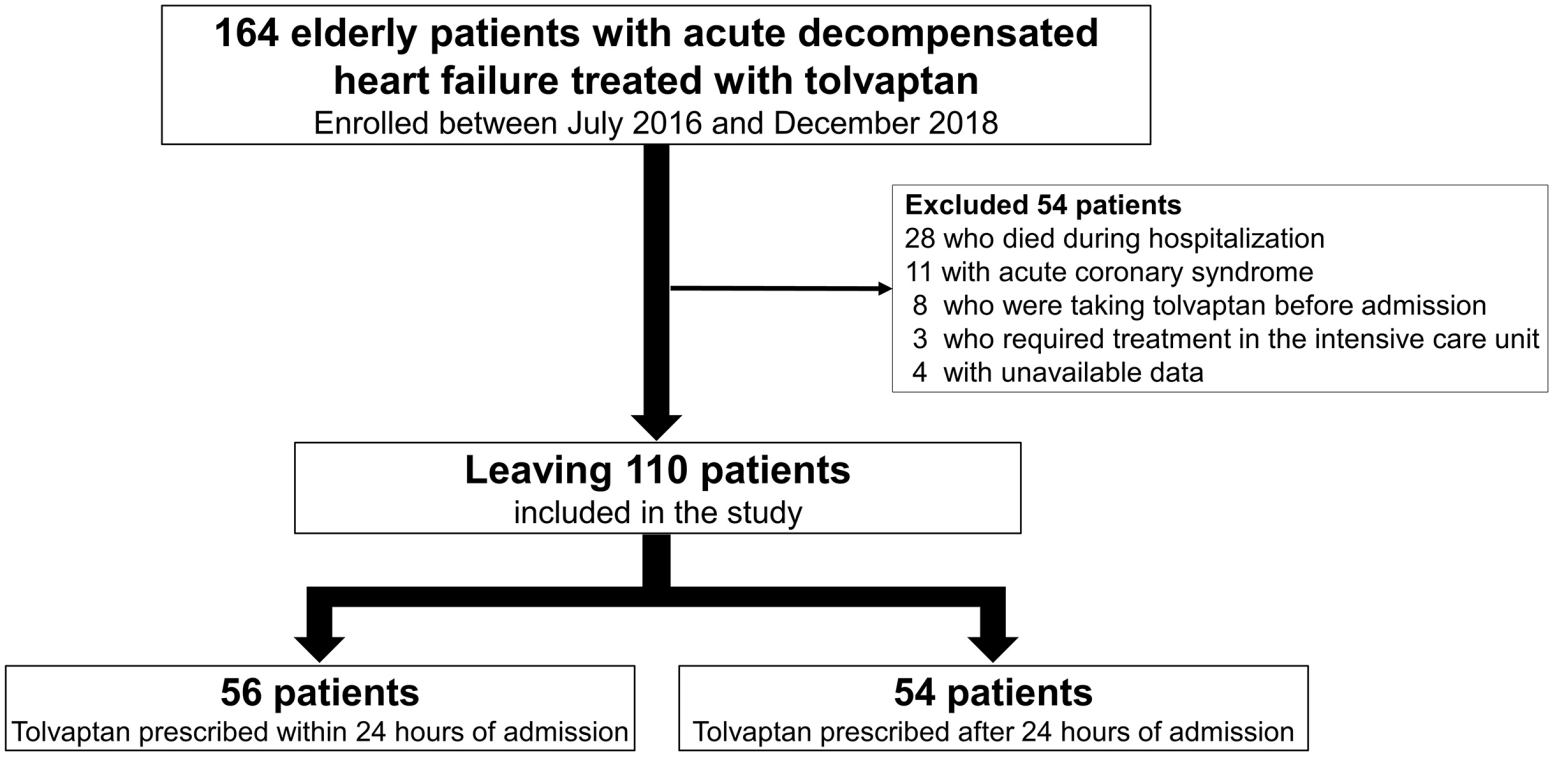
Patient flowchart.

This was a retrospective, single-center cohort study. The cohort initially registered elderly patients (age ≥ 75 years) admitted to Shinonoi General Hospital between July 2016 and December 2018 with a primary diagnosis of acute decompensated HF treated with tolvaptan. We excluded 28 patients who died during hospitalization, 11 who had acute coronary syndrome, 8 who had already taken tolvaptan before admission, 3 who required treatment in the intensive care unit, and 4 who had unavailable data. The applicability of the exclusion criteria was determined according to the medical records in our hospital. Data from the remaining 110 patients were included in our study (Figure 1). Informed consent was obtained using the Shinonoi General Hospital website; patients may decide to opt out anytime during the study process. The ethics committee of the Shinonoi General Hospital approved the study protocol (approval number 005). We collected data on clinical characteristics, medical history, major risk factors for HF, comorbidities, laboratory tests, electrocardiography, echocardiography, available angiographic data, acute management at admission, treatment and clinical events during hospitalization, and medications. Patients were then divided into two groups according to the median duration of admission to tolvaptan administration: those who received tolvaptan within 1 day (24 h) after admission (early treatment group) and those for whom tolvaptan was prescribed after 1 day (24 h) or more from hospitalization (add-on group). The length of hospital stay was the primary outcome. We compared the length of hospital stay between the two groups and investigated the relationship between early tolvaptan administration and length of hospital stay. The dosage of tolvaptan, the length of prescription, and the rate of continuous prescription at discharge were recorded. Acute decompensated HF was defined by the Framingham criteria ^(13)^, and the diagnosis of acute coronary syndrome was made by the treating clinicians using all available symptom, laboratory, electrocardiography, echocardiography, and coronary angiographic data. All data were fully anonymized before access. The investigation is consistent with the principles outlined in the Declaration of Helsinki.

### Statistical analysis

Continuous variables are summarized as the mean ± standard deviation if normally distributed and as the median and interquartile range if non-normally distributed. Normality was assessed by the Shapiro-Wilk test. Comparisons of baseline characteristics were made with a contingency table and the Pearson χ^2^ test for categorical variables, the *t*-test for normally distributed continuous variables, and the Mann-Whitney U test for non-normally distributed continuous variables. Spearman’s rank correlation method was used as a nonparametric measure of association between early tolvaptan use and clinical indices. Multivariable regression analysis was used to estimate the partial regression coefficient values and confidence interval values. Age, sex, serum creatinine, B-type natriuretic peptide, continuous dobutamine, and whether they live alone were the covariates selected, which were clinically considered to be associated with prolonged hospital stay in elderly patients. Linear regression analysis was used to assess the relationship between time of tolvaptan administration and length of hospital stay. A p-value of <0.05 was considered statistically significant. All statistical analyses were performed using SPSS Statistics software for Windows Version 26 (IBM Corp., Armonk, NY, USA).

## Results

### Baseline characteristics

Table 1 shows baseline characteristics of patients. The median age was 85 [interquartile range: 79-90] years, and 45% (n = 49) were female. Of the 110 patients enrolled in this study, 56 (51%) patients received tolvaptan within 1 day (24 h) after admission. Tolvaptan was administered at a median of 8 [4-15] days after admission in the add-on group. The mean dosage of prescribed tolvaptan was 5.9 ± 2.2 mg, and the median prescription length was 9 [4-21] days. Seventy-two patients (65%) took oral loop diuretics at admission. The median oral furosemide dose at admission was 30 [20-40] mg/day (Table 1). Intravenous furosemide administered at admission was more frequent in the add-on group than in the early treatment group. There were no other significant differences in baseline characteristics between the two groups, including serum creatinine. After analysis, there were no strong correlations between early tolvaptan and clinical indices (Table 2). At discharge, the median body weight decreased from 55 [46-66] kg to 50 [42-61] kg in the entire cohort. There was no difference in body weight reduction between the early tolvaptan group and the add-on group (−4.0 [−8.0, −2.0] kg vs. −5.5 [−8.3, −2.8] kg, p = 0.567). Forty-five (41%) patients continued tolvaptan after discharge (18 patients in the early tolvaptan group and 27 in the add-on group).

**Table 1.** Baseline Characteristics.

Variable	Overall population	Tolvaptan		p-value
		Early treatment	Added-on	
	(n = 110)	(n = 56)	(n = 54)	
Age (years)	85 [79-90]	84 [78-90]	88 [80-91]	0.235
Female, n (%)	49 (45)	24 (43)	25 (46)	0.717
BMI (kg/m^2^)	22.7 [20.4-26.1]	22.7 [20.5-26.1]	22.6 [20.1-26.3]	0.856
BW at admission (kg)	55 [46-66]	56 [46-67]	55 [48-65]	0.849
BW at discharge (kg)	50 [42-61]	51 [42-59]	50 [42-61]	0.815
BW change during hospitalization (kg)	−4.0 [−8.0, −2.0]	−4.0 [−8.0, −2.0]	−5.5 [−8.3, −2.8]	0.567
Systolic blood pressure (mmHg)	133 [114-159]	137 [121-160]	129 [111-156]	0.278
Diastolic blood pressure (mmHg)	82 [65-95]	81 [64-94]	82 [66-96]	0.756
LVEF (%)	55 [36-64]	59 [42-64]	54 [34-64]	0.528
LVEF ≥50%, n (%)	68 (62)	36 (64)	32 (59)	0.587
Previous HF admission, n (%)	31 (28)	15 (27)	16 (30)	0.740
Dilated cardiomyopathy, n (%)	12 (11)	5 (9)	7 (13)	0.497
Ischemic heart disease, n (%)	26 (24)	15 (27)	11 (20)	0.429
Hypertension, n (%)	72 (66)	34 (61)	38 (70)	0.287
Diabetes mellitus, n (%)	32 (29)	16 (29)	16 (30)	0.903
Chronic kidney disease, n (%)	50 (46)	26 (46)	24 (44)	0.835
Atrial fibrillation, n (%)	69 (63)	38 (68)	31 (57)	0.257
COPD, n (%)	8 (7)	4 (7)	4 (7)	0.957
Stroke, n (%)	32 (29)	15 (27)	17 (32)	0.588
Dementia, n (%)	30 (27)	16 (29)	14 (26)	0.755
Living alone, n (%)	10 (9)	6 (11)	4 (7)	0.482
**Oral diuretics at admission**
Oral furosemide, n (%)	57 (52)	30 (54)	27 (50)	0.708
Oral furosemide dose (mg/day)	30 [20-40]	40 [20-40]	20 [20-40]	0.556
Oral azosemide, n (%)	19 (17)	11 (20)	8 (15)	0.503
Oral azosemide dose (mg/day)	30 [30-60]	30 [30-60]	60 [15-105]	0.395
**Treatment at admission**
Continuous dobutamine, n (%)	36 (33)	15 (27)	21 (39)	0.176
Intravenous furosemide, n (%)	82 (75)	33 (59)	49 (91)	<0.001
Intravenous furosemide dose (mg/day)	20 [20-20]	20 [20-20]	20 [20-40]	0.003
**Laboratory data**
Hb (g/dL)	11.7 ± 2.4	12.0 ± 2.0	11.5 ± 2.8	0.327
ALB (g/dL)	3.4 ± 0.5	3.4 ± 0.4	3.4 ± 0.5	0.284
Serum creatinine (mg/dL)	1.19 [0.93-1.69]	1.18 [0.90-1.82]	1.19 [0.95-1.64]	0.759
Serum sodium (mEq/L)	140 [138-143]	140 [138-143]	141 [138-144]	0.344
Serum potassium (mEq/L)	4.4 ± 0.7	4.4 ± 0.7	4.4 ± 0.7	0.847
BNP (pg/mL)	731 [438-1378]	747 [465-1412]	727 [400-1353]	0.813
**Medication at discharge**
ACE-I/ARBs, n (%)	77 (70)	37 (66)	40 (74)	0.360
Beta-blockers, n (%)	70 (64)	36 (64)	34 (63)	0.885
MRAs, n (%)	62 (56)	31 (55)	31 (57)	0.828
Loop diuretics, n (%)	87 (79)	41 (73)	46 (85)	0.123

Values are presented as the mean ± SD, median [interquartile range], or n (%). Abbreviations: ACE-I, angiotensin-converting enzyme inhibitor; ALB, serum albumin; ARB, angiotensin-receptor blocker; BMI, body mass index; BNP, B-type natriuretic peptide; BW, body weight; COPD, chronic obstructive pulmonary disease; Hb, hemoglobin; HF, heart failure; LVEF, left ventricular ejection fraction; MRA, mineralocorticoid receptor antagonist.

**Table 2.** Univariate Spearman’s Rank Correlations between Early Tolvaptan and Clinical Indices.

Variable	Spearman’s r	p-value
Age (years)	−0.114	0.236
Sex	0.035	0.720
Serum creatinine (mg/dL)	−0.029	0.761
BNP (pg/mL)	0.023	0.814
Continuous dobutamine	−0.129	0.179
Living alone	0.072	0.487

Abbreviations: BNP, B-type natriuretic peptide

### Clinical outcomes

**Figure 2.**
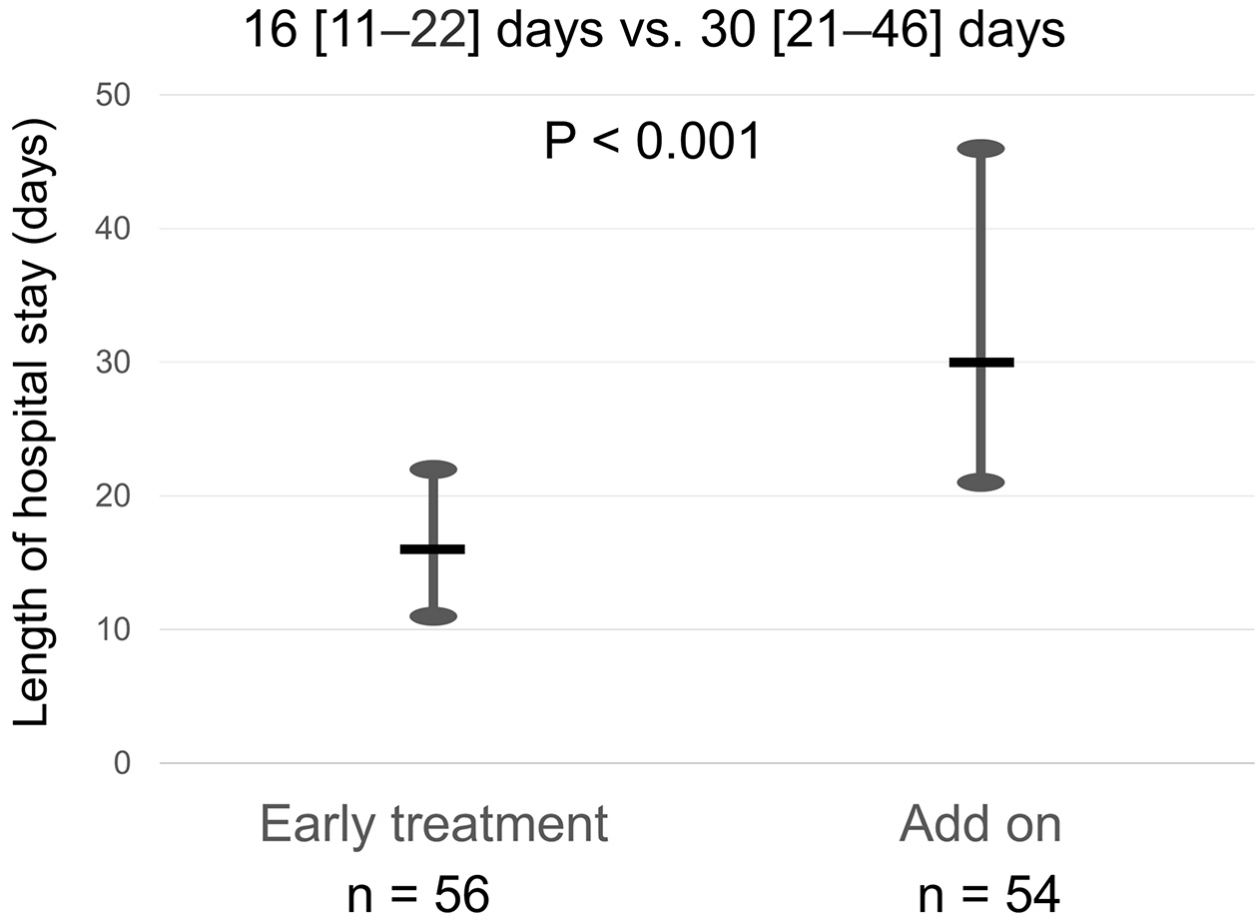
Length of hospital stay in relation to the time of tolvaptan administration - the early treatment group had a significantly shorter length of hospital stay than the add-on group.

**Figure 3.**
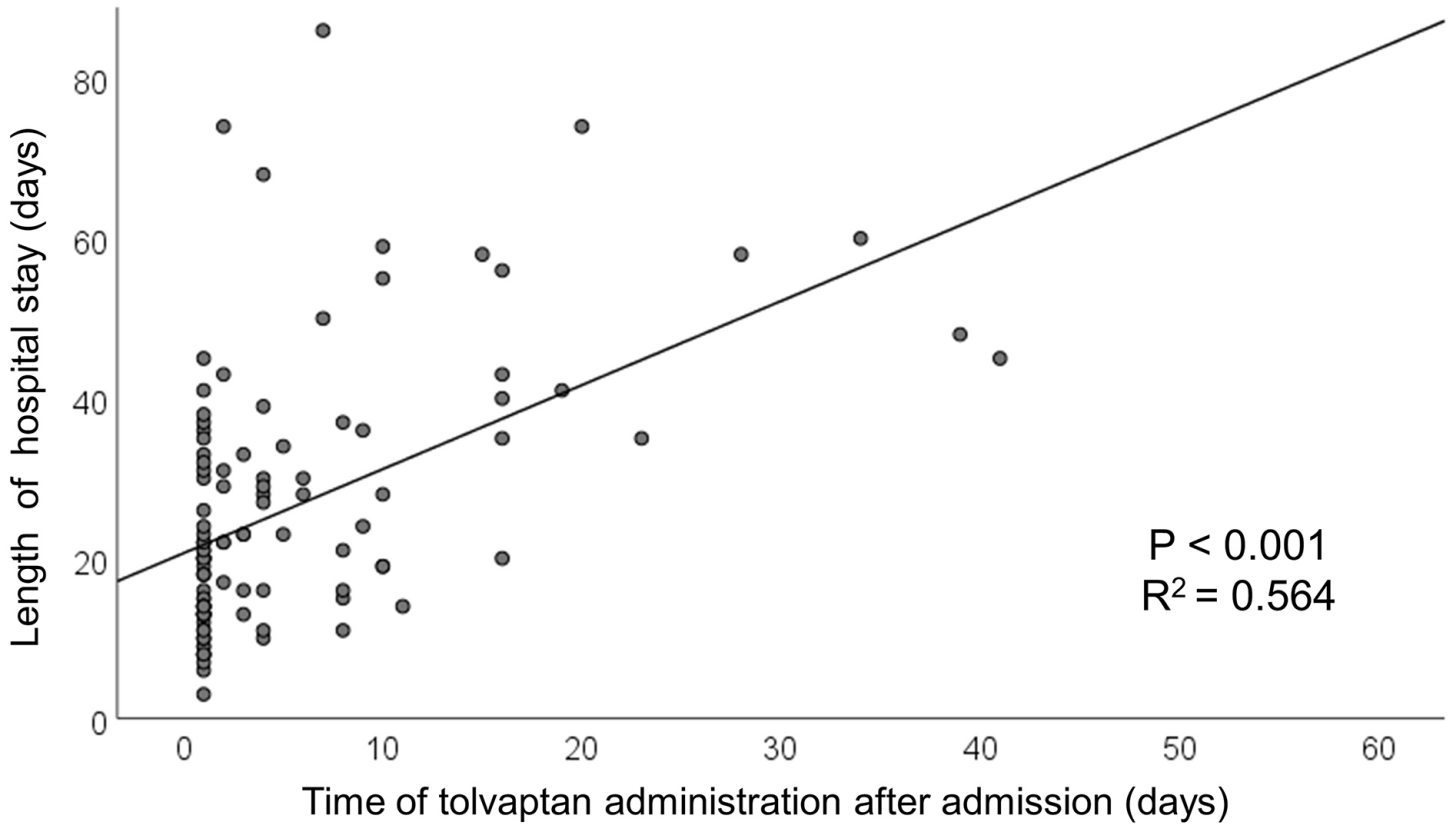
Linear regression analysis of the relationship between time of tolvaptan administration and length of hospital stay - linear regression analysis showed a positive relationship between time of tolvaptan administration and length of hospital stay.

The median length of hospital stay was 22 [14-35] days. The early treatment group had a significantly shorter length of hospital stay than the add-on group (16 [11-22] days vs. 30 [21-46] days, p < 0.001, Figure 2). On multivariable regression analysis, early tolvaptan administration was associated with reduced length of hospital stay after adjusting for age, sex, serum creatinine, B-type natriuretic peptide, continuous dobutamine, and whether they live alone (partial regression coefficient −16.213 [−24.132, −8.295], p < 0.001) (Table 3). Linear regression analysis showed a positive relationship between time of tolvaptan administration and length of hospital stay (R2 = 0.564, p < 0.001) (Figure 3).

**Table 3.** Multivariable Regression Analysis According to Length of Hospital Stay.

Variable	Partial regression coefficient (95% CI)	p-value
Early tolvaptan treatment	−16.213 (−24.132, −8.295)	<0.001
Age (years)	0.264 (−0.263, 0.791)	0.321
Male (sex)	−10.634 (−19.404, −1.864)	0.018
Serum creatinine (mg/dL)	5.955 (−0.735, 12.645)	0.080
BNP (pg/mL)	0.004 (−0.001, 0.008)	0.088
Continuous dobutamine	3.017 (−5.606, 11.639)	0.489
Living alone	−4.377 (−17.995, 9.241)	0.524

Abbreviations: BNP, B-type natriuretic peptide

## Discussion

The novel finding of this study is that early administration of tolvaptan was significantly associated with a reduced length of hospital stay in elderly patients with acute decompensated HF. Moreover, linear regression analysis revealed a positive relationship between time of tolvaptan administration and length of hospital stay among this group of patients.

In the EVEREST trial, tolvaptan was not found to improve the long-term prognosis of patients with acute decompensated HF ^(14)^. However, several studies have reported that tolvaptan reduces renal injury, which may beneficially affect their prognosis ^(15), (16)^. In addition, other studies have reported the beneficial effects of early tolvaptan treatment in HF patients such as increased weight loss ^(9)^, reduced WRF ^(11), (17)^ or in-hospital death ^(10)^, and improved mid-term prognosis ^(11), (17)^.

This study demonstrated a significant association between early administration of tolvaptan and reduced length of hospital stay among elderly Japanese patients hospitalized for acute decompensated HF. To our knowledge, this study is the first to investigate the administration of tolvaptan within 1 day (24 h) of admission in relation to length of hospital stay in such patients. There were no significant differences in baseline characteristics between the early treatment group and the add-on group. Our findings have important clinical implications that lead us to suggest that early administration of tolvaptan is a feasible approach to shorten the length of hospital stay. Furthermore, shortening the length of hospital stay may have the possibility to maintain ADL and QOL in elderly patients with HF.

Tolvaptan is additionally administrated a few days after admission in many cases, when the resistance to loop diuretics becomes apparent. The approved indications of tolvaptan and its drug prices may be reasons why the prescription of tolvaptan is limited in the acute phase after admission. However, the results of recent studies ^(10), (11), (12), (17)^ and this study suggest that the optimal timing of administration may be earlier than currently considered (e.g., within the next 24 h if the furosemide bolus was refractory in acute decompensated HF).

Our findings suggest that early administration of tolvaptan could be a treatment option in elderly patients with acute decompensated HF if the initial diuretic response remains inadequate. By administering tolvaptan earlier in patients with initial diuretic resistance, patients may be discharged in a shorter period, which might lead to maintaining their ADL and QOL.

Our study has some limitations. First, we included only a small number of extremely elderly patients taken from a single-center. The consecutive patients enrolled in this study represent a highly selected cohort. Further randomized controlled trials and observational studies involving larger numbers of patients are needed to verify our hypothesis. Second, we could not evaluate the response to tolvaptan in this study. Third, there was no fixed protocol regarding the administration of tolvaptan. The dosing and timing differed between patients and attending physicians, and the initial urine output was not accurately considered. According to clinical decisions, some patients were prescribed tolvaptan without intravenous loop diuretics (all treated with oral loop diuretics). One of the reasons for this limitation could be the study period when optimal dosing, timing, and methods were still not established ^(18), (19)^, whereas the recent guidelines from Western countries mention specific diuretic therapy for acute HF ^(20), (21)^. Finally, although there were no significant differences in baseline characteristics between the two groups, confounding factors could have affected the results.

In conclusion, early tolvaptan administration was associated with a reduced length of hospital stay in elderly patients hospitalized for acute decompensated HF. Early administration of tolvaptan could be a treatment option in these patients if the initial diuretic response remains inadequate.

## Article Information

### Conflicts of Interest

SS has received lecture fees from Otsuka Pharmaceutical Co., Ltd.

KK^†^ has received lecture fees from Astellas Pharma Inc., AstraZeneca K.K., MSD K.K., Otsuka Pharmaceutical Co., Ltd., Ono Pharmaceutical Co., Ltd., Kyowa Kirin Co., Ltd., Kowa Co., Ltd., Sanofi K.K., Sumitomo Dainippon Pharma Co., Ltd. (Sumitomo Pharma Co., Ltd.), Mitsubishi Tanabe Pharma Corp., Eli Lilly Japan K.K., Nippon Boehringer Ingelheim Co., Ltd., Novartis Pharma K.K., Novo Nordisk Pharma Ltd., Bayer Yakuhin, Ltd., Pfizer Japan Inc., and Janssen Pharmaceutical K.K. and funded research or joint research expenses from Kowa Co., Ltd., AstraZeneca K.K., Daiichi Sankyo Co., Ltd., Novo Nordisk Pharma Ltd., Amgen, Janssen Pharmaceutical K.K., Parexel International Inc., and Astellas Pharma Inc. His affiliated institution (Shinshu University School of Medicine) has received grants from Otsuka Pharmaceutical Co., Ltd., Mitsubishi Tanabe Pharma Corp., Nippon Boehringer Ingelheim Co., Ltd., and Kyowa Kirin Co., Ltd., and his department has endowed chairs from Medtronic Japan Co. Ltd., Boston Scientific Japan K.K., Abbott Japan LLC, Japan Lifeline Co., Ltd., Biotronik Japan, Terumo Corporation, Nipro Corporation, and Cordis Japan G.K.

The other authors declare no conflicts of interest.

### Author Contributions

Conceptualization: SS, KK

Data curation: SS, NY, AF

Formal analysis: SS

Funding acquisition: none

Investigation: SS

Methodology: SS, KK, HM

Project administration: SS, AF

Resource: none

Software: none

Supervision: KK, NY, AF, YK, TM, NH, AK, HM, KY, KK^†^

Validation: SS

Visualization: SS, KK, NY

Writing - original draft: SS, KK

Writing - review and editing: SS, KK, NY, AF, YK, TM, NH, AK, HM, KY, KK^†^

KK = Kazuhiro Kimura

KK^†^ = Koichiro Kuwahara

Sho Suzuki and Kazuhiro Kimura contributed equally to this work.

### Approval by Institutional Review Board (IRB)

Minaminagano Medical Center, Shinonoi General Hospital, No. 005
